# Bragg-Grating-Based Photonic Strain and Temperature Sensor Foils Realized Using Imprinting and Operating at Very Near Infrared Wavelengths

**DOI:** 10.3390/s18082717

**Published:** 2018-08-18

**Authors:** Jeroen Missinne, Nuria Teigell Benéitez, Marie-Aline Mattelin, Alfredo Lamberti, Geert Luyckx, Wim Van Paepegem, Geert Van Steenberge

**Affiliations:** 1 Center for Microsystems Technology (CMST), Ghent University and imec, 9052 Ghent, Belgium; nuria.teigellbeneitez@ugent.be (N.T.B.); MarieAline.Mattelin@ugent.be (M.-A.M.); Geert.Vansteenberge@ugent.be (G.V.S.); 2 Department of Materials, Textiles and Chemical Engineering (MaTCh), Ghent University, 9052 Ghent, Belgium; Alfredo.Lamberti@ugent.be (A.L.); Geert.Luyckx@ugent.be (G.L.); Wim.VanPaepegem@ugent.be (W.V.P.)

**Keywords:** Bragg grating sensor, flexible sensor, foil, nanoimprint lithography, Ormocer, polymer, strain gage rosette, strain sensor, temperature sensor, waveguide

## Abstract

Thin and flexible sensor foils are very suitable for unobtrusive integration with mechanical structures and allow monitoring for example strain and temperature while minimally interfering with the operation of those structures. Electrical strain gages have long been used for this purpose, but optical strain sensors based on Bragg gratings are gaining importance because of their improved accuracy, insusceptibility to electromagnetic interference, and multiplexing capability, thereby drastically reducing the amount of interconnection cables required. This paper reports on thin polymer sensor foils that can be used as photonic strain gage or temperature sensors, using several Bragg grating sensors multiplexed in a single polymer waveguide. Compared to commercially available optical fibers with Bragg grating sensors, our planar approach allows fabricating multiple, closely spaced sensors in well-defined directions in the same plane realizing photonic strain gage rosettes. While most of the reported Bragg grating sensors operate around a wavelength of 1550 nm, the sensors in the current paper operate around a wavelength of 850 nm, where the material losses are the lowest. This was accomplished by imprinting gratings with pitches 280 nm, 285 nm, and 290 nm at the core-cladding interface of an imprinted single mode waveguide with cross-sectional dimensions 3 × 3 µm^2^. We show that it is possible to realize high-quality imprinted single mode waveguides, with gratings, having only a very thin residual layer which is important to limit bend losses or cross-talk with neighboring waveguides. The strain and temperature sensitivity of the Bragg grating sensors was found to be 0.85 pm/µε and −150 pm/°C, respectively. These values correspond well with those of previously reported sensors based on the same materials but operating around 1550 nm, taking into account that sensitivity scales with the wavelength.

## 1. Introduction

Printing technologies have long been used in electronics owing to their fabrication flexibility, low cost, and short time-to-market, but also for photonic applications printing technologies start to find widespread use owing to superior resolution and fabrication quality of emerging printing technologies. For example, optical waveguides printed in glass using a tightly focused laser [1], optical coupling structures 3D printed using two-photon polymerization [2], and assembly of micro-optical components using laser transfer printing [3]. Also, printed electrical connection technologies are becoming increasingly precise so that they can be used for alignment-critical (electro-)optical assemblies. The abovementioned laser transfer printing technique can be used to precisely deposit solder bumps for fine-pitch photodiode or Vertical-Cavity Surface-Emitting Laser (VCSEL) array chips [4]. Furthermore, interconnections between electro-optical transmitter/receiver chips and drivers are traditionally provided by wirebonds, but this technology is reaching its limits for very high-speed applications and the wire loops prevent optical fibers from probing features nearby the wirebonds. Aerosol jet printing has therefore recently been proposed as a promising alternative to replace wirebonds for these high-speed applications [5]. Finally, optical sensors, such as ring resonators [6] or liquid crystalline film-based sensors [7] have been inkjet printed.

The current paper will focus on another type of printing technology, namely a stamp-based imprinting process which allows the definition of passive nano- or microphotonic structures with very good control over the dimensions of the printed features. This technology, mostly know as nanoimprint lithography (NIL), has been developed for patterning nanometer-sized features well-beyond the resolution limits of standard lithography [8]. Many variations on the basic concept have been reported, but especially the imprinting approaches in which soft polymeric stamps are used [9] are interesting for the printing industry because they allow conformal imprinting on large areas, easy releasing of the stamp, and eventually allow roll-to-roll [10,11] or roll-to-plate high-throughput manufacturing [12]. Apart from a larger range of applications in the electronics industry, the imprinting technology has also been employed to fabricate optical nanostructures such as photonic crystals [9] or gratings [13], but the technology is also suitable for defining features with larger dimensions, such as polymer-based ring-resonators [14,15], evanescent wave sensors [16], and optical interconnects [17].

Herein, we will report on an imprinting process suitable for realizing Bragg-grating-based optical sensors on foil, for multi-axial strain and temperature sensing. A Bragg grating is a periodic structure, applied in an optical waveguide; when this structure is excited with a broad spectrum, it reflects a single wavelength, called the Bragg wavelength (*λ_B_*). This can be exploited as a sensor since the reflected wavelength is very sensitive to environmental conditions imposed, such as strain or temperature. Bragg grating sensors in optical fibers have since long been used, mainly for strain sensing [18]. However, those sensors are mainly sensitive in the direction along the fiber, and therefore, multiple sensors in precisely defined orientations are required to record the complete strain field in a certain plane. As an alternative, we have previously shown that it is possible to realize such Bragg grating sensors in thin flexible foils, making it possible to implement multiple closely spaced sensors in well-defined directions [19], as such enabling realizing an optical variant of electrical strain gage rosettes. Such a strain gage rosette consists for example of three sensors, each angularly displaced by 45°, and is very suitable for measuring the strains relative to the material-symmetry axes regardless of the orientation of the sensor foil on the surface. This previous work was focused on Bragg grating sensors operating at the telecom wavelength range, since a large variety of standard sensor readout equipment is available because commercial fiber Bragg gratings mainly operate in this wavelength range. Furthermore, fabrication of the gratings is easier at longer wavelengths because of the larger pitches required.

The novelty of the current paper is the development of sensor foils operating in the very near-infrared wavelength range around 850 nm, for several reasons. Firstly, the optical material losses of the available optical polymers are the lowest in this wavelength range, which potentially allows longer waveguides in which more sensors can be multiplexed. Secondly, we have previously shown that it is possible to realize an ultra-thin and compact Bragg grating based sensing system completely integrated with sources and detectors [20]. This approach requires suitable VCSEL chips, which are currently not yet largely available at telecom wavelengths. Finally, sensors operating below a wavelength of 1000 nm are compatible with largely available and low-cost Si-based detectors.

## 2. Sensor Design

### 2.1. Concept and Layout of the Multi-Axial Photonic Strain Sensor

**Figure 1:**
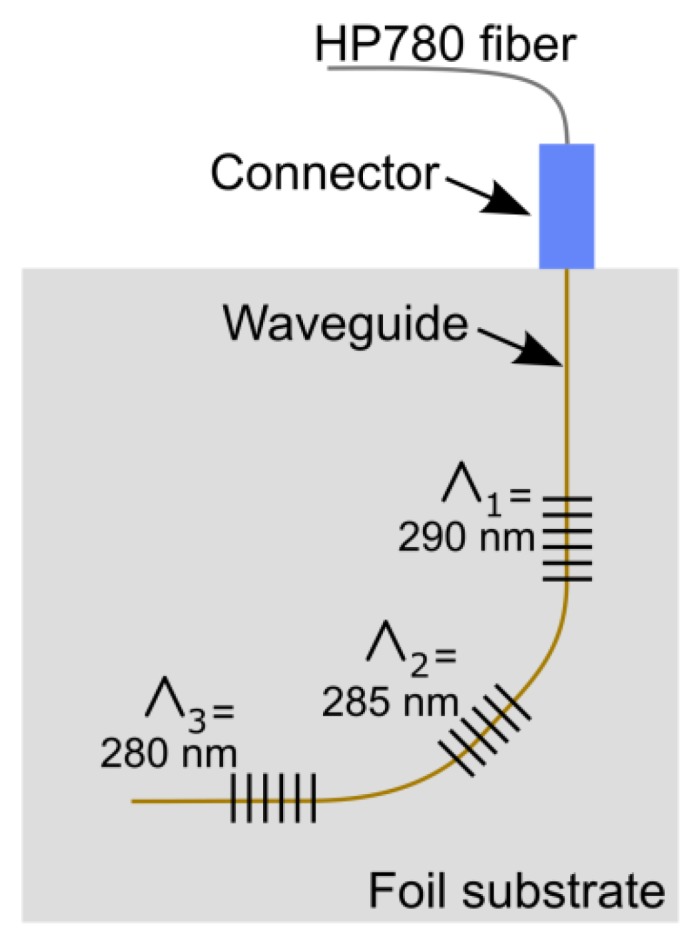
Layout of the strain sensor rosette showing the orientation of the grating sensors and the waveguide running perpendicularly over the gratings.

The Bragg wavelength *λ_B_*, in case of a first order grating, depends on the grating pitch *Λ* and the effective refractive index of the mode propagating in the waveguide (*n_eff_*), so that *λ_B_ = 2n_eff_Λ*. This means that multiple gratings with slightly different pitch can be multiplexed in the same waveguide so that their Bragg wavelengths are separated by several nanometer avoiding spectral cross-talk between sensors. An optical strain gage rosette, using a single, bent waveguide, can therefore be realized as schematically illustrated in Figure 1. The detailed optical design of the waveguide and the grating sensors is discussed in the following subsections.

### 2.2. Waveguide Design for Single Mode Operation around λ = 850 nm

The waveguide was realized in OrmoCore and OrmoClad (MicroResist Technology, Berlin, Germany), both Ormocer®-based hybrid organic-inorganic materials and was optimized using a numerical mode solver, taking into account the material refractive indices (RI), and the requirement that the waveguides should be single mode at the operation wavelength with a mode field diameter (MFD) as close as possible to the mode field diameter of the single mode optical fiber (HP780 fiber, MFD = 5.0 ± 0.5 µm at *λ* = 850 nm) used to read out the sensor. This ensures a good coupling efficiency between the fiber and the waveguide on the sensor foil and minimizes the bend losses [21]. An advantage of the used materials, OrmoCore (RI = 1.54 at *λ* = 850 nm, MicroResist Technology, Berlin, Germany) and OrmoClad (RI = 1.525 at *λ* = 850 nm, MicroResist Technology, Berlin, Germany), is that they can be mixed, and depending on the mixing ratio, a continuous range of RI values between the RI values of the pure materials can be obtained, providing an additional degree of freedom for the waveguide design. Based on these criteria, a waveguide with a cross-sectional dimension of 3 × 3 µm^2^ was selected where the core is formed by pure OrmoCore and the cladding consists of a 1:1 mixture (by weight) OrmoCore:OrmoClad (RI = 1.533). For these waveguide dimensions, the MFD was simulated to be 4.7 µm with an effective index of the mode being 1.535.

### 2.3. Bragg Grating Sensor Design

Because it is intrinsically challenging to define the small features required for sensor operation around 850 nm, some of the grating design parameters were chosen to relax the fabrication process as much as possible. The duty cycle of the grating was chosen to be 50% and the grating depth 120 nm to limit the aspect ratio of the grating features. Since the reflectivity of the grating is mainly determined by its depth and number of periods, the latter parameter was chosen long enough (1 cm) to ensure sufficient reflectivity. The spectral reflection response, *R*, of a rectangular grating with depth *a* can be simulated based on coupled-mode theory [22], using the following equation:

(1) $$R={\left|\frac{j\kappa \;\sin\left(\sqrt{\delta^{2}-\kappa^{2}}\;L\right)}{\sqrt{\delta^{2}-\kappa^{2}}\cos\left(\sqrt{\delta^{2}-\kappa^{2}}\;L\right)-j\delta \sin\left(\sqrt{\delta^{2}-\kappa^{2}}\;L\right)}\right|}^{2},$$

with *κ* the coupling coefficient described in Equation (2), *δ* defined by Equation (3), and *L* the length of the grating.

(2) $$\kappa =\;\frac{2\pi^{2}a^{3}}{3\lambda_{B}ld^{3}}\;\frac{n_{c}^{2}-\;n_{cl}^{2}}{n_{c}^{2}}\;\left[1+\;\frac{3\lambda_{B}}{2a\pi {\left(n_{c}^{2}-\;n_{cl}^{2}\right)}^{0.5}}+\;\frac{3\lambda_{B}^{2}}{4a^{2}\pi^{2}\left(n_{c}^{2}-n_{cl}^{2}\right)}\right]$$

(3) $$\delta =n_{eff\;}\left(\frac{2\pi }{\lambda }-\frac{2\pi }{\lambda_{B}}\right)$$

In these equations, *l* is the grating order, which is 1; *n_c_* and *n_cl_* are the refractive indices of the core and cladding, respectively; *n_eff_* is the effective refractive index of the propagating waveguide mode; and *d* the cross-sectional dimension of the waveguide (3 µm). 

**Figure 2:**
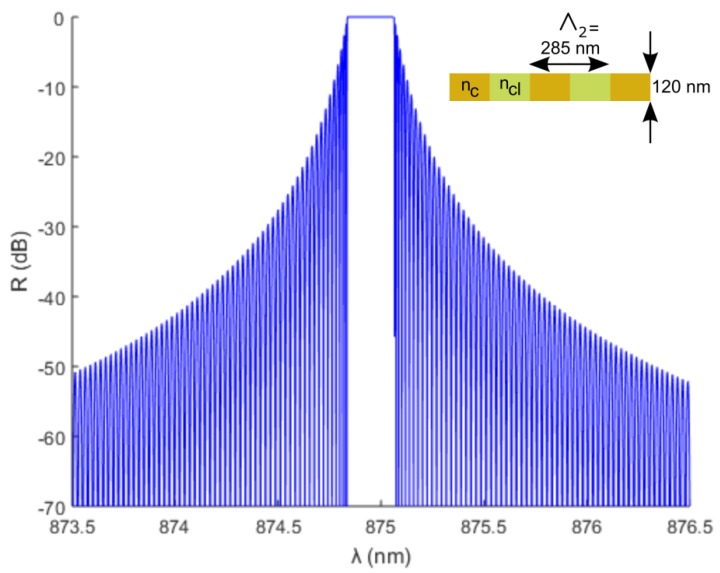
Calculated reflection spectrum for grating sensor 2 with dimensions as shown in the inset.

In Figure 2, a calculated reflection spectrum is shown for a Bragg grating with a pitch of 285 nm (sensor 2), duty cycle 50%, depth 120 nm, and grating length 1 cm. The peak 3 dB bandwidth is 0.245 nm for sensor 2. For the other sensors the pitch is either 5 nm smaller or larger, which results in a shift of the spectrum and a slightly different 3 dB bandwidth, as summarized in Table 1.

## 3. Methods

### 3.1. Sensor Fabrication

**Figure 3:**
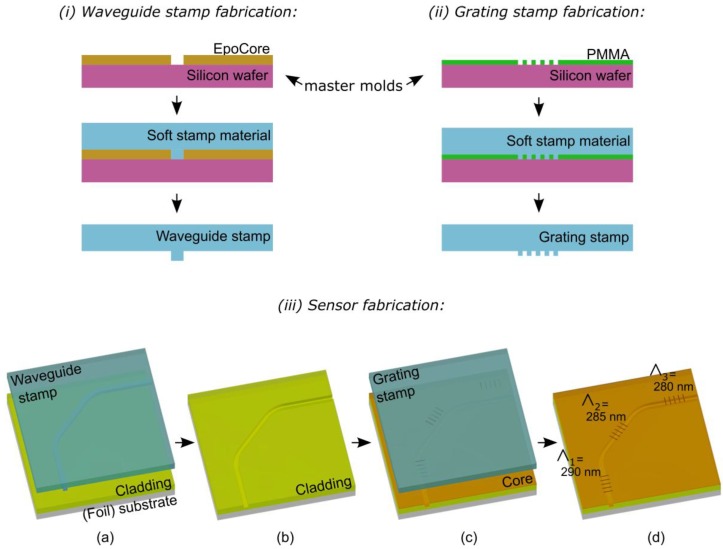
Process flow for the imprinting of waveguides with grating sensors.

The Ormocer®-based waveguide Bragg grating sensors were realized on a 175 µm thick Polyethylene terephthalate (PET) foil carrier employing imprinting lithography for patterning the required micro-and nanostructures. This technique consists of bringing a stamp with the required patterns in contact with the liquid and UV-curable Ormocer^®^ layers. In our case, the stamp is made from a transparent, soft polymer material so that the Ormocer^®^ can be UV cured while the stamp is in contact thereby permanently fixing the patterns in the material. Two imprinting steps are required to realize the sensor. A first imprinting step is used to define the single mode waveguides by imprinting a microchannel with the required dimensions in the polymer “cladding” material after which this channel is filled by spin-coating a polymer “core” material. A second imprinting step is used to define the grating sensors in this core material. Both imprinting steps require a suitable polymer soft stamp, which is in itself a replication of a so-called master mold. The process of fabricating the two master molds, the two soft stamps, and finally the flexible sensor foil is described in detail below and illustrated in Figure 3.

Firstly, a master mold for the waveguides was fabricated having microchannels with the required dimensions. Since the optical waveguides require cross-sectional dimensions of 3 × 3 µm^2^, standard lithography was used. Therefore, a 3 µm thick EpoCore_2 negative resist layer (MicroResist Technology, Berlin, Germany) was spin-coated (2000 rpm for 30 s) on a cleaned 4” silicon wafer. This negative resist material was chosen for its high resolution, stability, and capability to produce vertical sidewalls. After a soft-baking step (2 min at 50 °C and then 4 min at 90 °C), a photomask having 3 µm wide lines was brought in contact with this layer and UV-exposed with a dose of 200 mJ/cm^2^. After a post-baking step followed by a developing step to remove the unexposed regions, approximately 3 µm wide and 3 µm deep channels remain in the resist. The master mold for the very fine grating structures, on the other hand, was realized using e-beam lithography. Therefore, a 120 nm thick PMMA resist (950 PMMA A3, MicroChem, Westborough, MA, USA) was spin-coated (3000 rpm, 45 s) on a cleaned 4” silicon wafer. This e-beam resist was chosen for its high resolution and because optimized recipes for the required coating thickness were available in house. After a soft baking step (180 °C, 1 min), the wafer was loaded into a Raith Voyager e-beam lithography system and the grating lines were exposed with a dose of 350 µC/cm^2^. The exposed lines were removed during a developing step using a 3:1 IPA–MIBK solution.

Secondly, soft stamps were fabricated by replicating the master molds into a UV-curable transparent perfluoropolyether (PFPE) polymer. This polymer was prepared by adding 3% Irgacure 2022 photoinitiator (BASF, Antwerp, Belgium) to Fomblin MD 40 (Solvay, Brussels, Belgium) by weight. After manually mixing thoroughly, the viscous mixture was let to rest for 30 min for degassing. Subsequently, this mixture was spin-coated at a slow speed (500 rpm, 60 s) to achieve a relatively thick but homogeneous layer on the previously made master mold and afterwards it was covered using a PET tape with the sticky side touching the spin-coated layer. This stack was UV-exposed (30 mW/cm^2^, 30 s) and peeled off from the master mold once cured.

The first soft stamp was used to imprint the waveguides. As mentioned above, a 1:1 mixture of OrmoCore–OrmoClad was used as cladding material to achieve the required refractive index contrast. This mixture was spin-coated (3000 rpm, 30 s, coating thickness: 35 µm) on a 5 mm × 5 mm PET foil (PMX739, 175 µm thick, Hifi Film, Stevenage, UK) which was plasma treated (Diener Pico, 190 W 40 kHz generator, 24 s, 0.8 mbar, gas used: air; Diener electronic, Ebhausen, Germany). Then the stamp was brought in contact with the coating in a rolling motion to avoid air being trapped. This stack was UV-exposed (30 mW/cm^2^, 10 s) after which the soft stamp was manually peeled off and the cladding layer with imprinted channels was baked in an oven (120 °C, 90 min) to complete the polymerization process. Afterwards, the waveguide core was realized by spin-coating (3000 rpm, 30 s) a 1:2.5 Ormocore–maT-1050 (MicroResist Technology, Berlin, Germany) mixture by weight on the plasma treated (Diener Pico, 190 W 40 kHz generator, 24 s, 0.8 mbar, gas used: air) microchannel layer. The maT-1050 solvent was used to dilute the OrmoCore material, so that a thin coating, just filling the channels could be obtained. This solvent was evaporated during a soft baking step (100 °C, 5 min). Then, the second soft stamp (with the grating structures) was applied (manually) onto the OrmoCore layer ensuring that the gratings were aligned at the proper location with respect to the waveguides. To facilitate this manual alignment, large 1 cm^2^ grating islands were used on the soft stamp. This step imprints the grating sensors at the top surface of the waveguides, and at the same time ensures the capillary filling of the channels with the core material. The spin-coating thickness was optimized to have as little residue as possible between the channels while having enough material to completely fill the channels. While the stamp is in place, a UV exposure (30 mW/cm^2^, 15 s) was applied in a nitrogen chamber to cure the OrmoCore material. After removing the stamp, the layer was baked in an oven and a cladding layer was applied using the same parameters as for the bottom cladding layer, but without using the stamp for imprinting the channels. As such, a 3 × 3 µm^2^ waveguide with integrated grating sensors was achieved, which was completely surrounded and protected with cladding material.

### 3.2. Sensor Characterization Methods

The realized photonic foil sensors were characterized in terms of their strain and temperature sensitivity. Therefore, a dedicated fiber connector was applied onto the sensor foil to record the optical sensor signals. Furthermore, a readout system was developed to obtain the spectral sensor data.

#### 3.2.1. Readout System

**Figure 4:**
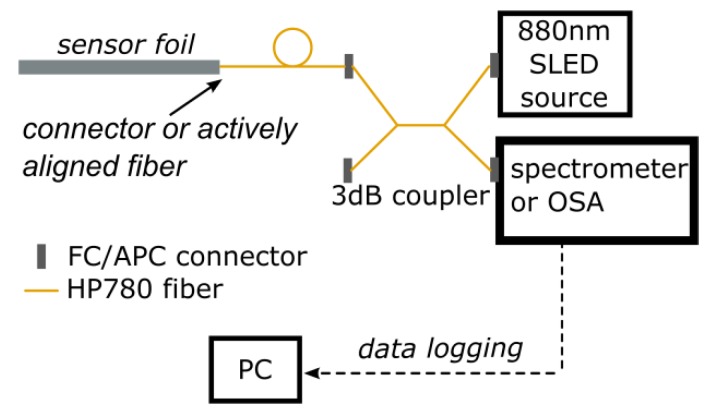
System for reading out Bragg grating sensor foils.

Commercial fiber Bragg grating sensors are read out using commercially available interrogators operating in the telecom wavelength range. Such devices allow recording the reflection spectrum of multiple Bragg grating sensors and tracking of the Bragg wavelength at a fast rate (typically at 100 Hz up to several kHz). However, interrogators operating in the near-infrared, around 850 nm, are not widespread. Because of this, and since standard optical spectrum analyzers (OSA) are rather slow and bulky and therefore difficult to combine with mechanical test setups, we chose to implement a compact and relatively fast readout system using an Ocean Optics USB spectrometer. A schematic of this readout system is shown in Figure 4. A broadband, superluminescent diode (SLED; Exalos (Schlieren, Switzerland) EXS210088-01, center wavelength 880 nm, bandwidth 70 nm) pigtailed to a single mode fiber was used as a source to illuminate the grating sensors, and with the use of a 2 × 2 3 dB splitter, the reflected signal was sent to the spectrometer. A Python script, running on a PC was then continuously reading out spectra through the USB connection and performing a peak detection. This system allowed tracking of the three sensor reflection peaks at about 100 Hz, which was sufficient for characterizing the sensor foils. For some experiments, where mentioned below, an OSA (Agilent 86142B, Santa Clara, CA, USA) was used for improved spectral resolution.

#### 3.2.2. Methods for Characterizing Strain Sensitivity

**Figure 5:**
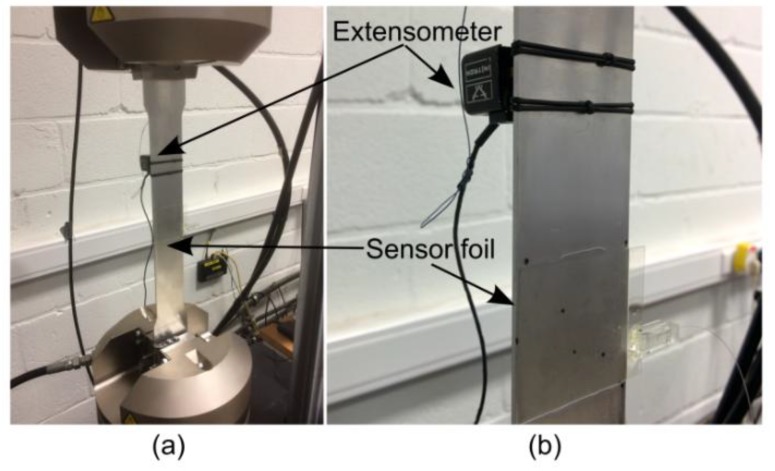
(**a**) Tensile test setup and (**b**) close-up view on the mounted sensor foil and extensometer.

A sensor foil containing three sensors in a rosette configuration was glued onto a 40 mm wide, 2 mm thick aluminum dogbone-shaped test specimen using a thin layer of 2-component epoxy glue (Loctite EA 9483, Düsseldorf, Germany). This test specimen was clamped into a servohydraulic Instron 8800 machine, and a tensile load was applied at a displacement rate of 0.05 mm/min until a maximum strain of 1000 µε was achieved, while the wavelength shift of all three sensors was continuously recorded using the readout system. As a reference, the applied strain was also recorded using an extensometer, placed above the sensor foil, on the aluminum test specimen. Figure 5 shows how the dogbone specimen was clamped and shows the position of the sensor foil and the extensometer.

To verify whether the sensor sensitivity is the same in compression and in tension, an additional cantilever deflection test was performed. The same aluminum test specimen with attached sensor was therefore mounted in a horizontal position and clamped on 1 side, while an increasing vertical deflection was applied on the other side by pushing it down using a motorized translation stage. This experiment was performed both with the sensor facing up (loading in tension) and down (loading in compression), with the same loading conditions. In both cases, the wavelength shift experienced by the sensor parallel to the long axis of the test specimen was recorded as a function of the applied deflection.

#### 3.2.3. Methods for Characterizing Temperature Sensitivity

The temperature sensitivity was assessed by heating a free-standing sensor foil on a hotplate and recording the Bragg wavelength as a function of temperature. The accuracy on the determined sensitivity will depend on the accuracy of the measured reference temperature at the grating location. Therefore, a thermocouple was placed directly on top of each grating sensor as a more reliable reference compared to reading the hotplate setpoint. A good contact between the thermocouples and the sensor foil was ensured by covering them with a glass plate held in place by a 100 g metal block. During the experiment, an external HP780 optical fiber was actively realigned with the optical waveguide in the foil each time a stable temperature was reached. Then, the reflection spectrum was acquired using an OSA (spectral resolution was set at 200 pm) and the Bragg wavelength shift of the sensors as a function of the temperature measured by the thermocouples was determined.

## 4. Results and Discussion

### 4.1. Imprinted Waveguides

**Figure 6:**
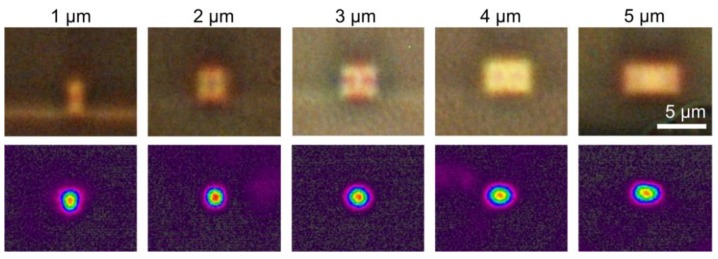
Cross-sectional microscope images of 3 µm thick imprinted waveguides having different target widths (as mentioned in the column header) and corresponding mode field profiles imaged at the waveguide end-face (*λ* = 850 nm).

The fabrication process was validated by inspecting the cross-sections of waveguides having different widths (constant height of 3 µm) using a microscope, see Figure 6. The middle column corresponds with the waveguide used for realizing the sensors (cross-sectional dimensions 3 × 3 µm^2^). Since the refractive index contrast between the cladding material (OrmoClad–OrmoCore 1:1 mix) and the core material (OrmoCore) is very limited (0.007) and the waveguide structures were very small, backside illumination in combination with a high magnification objective was needed to visualize the core, making it more difficult to obtain sharp images. However, the figure clearly shows well-defined waveguide structures with very small residual layer on both sides of the core. Although the exact thickness of this residual layer could not be determined because of the limited spatial resolution of microscopy, it can be estimated well below 1 µm based on the cross-sectional images. To further optically characterize the waveguides, light (*λ* = 850 nm) was launched (using an HP780 single mode fiber, Thorlabs, Newton, NJ, USA) into a straight waveguide section from 1 side, and the mode profile was imaged at the other side using a near-field beam profiler (Spiricon SP620U in combination with a near-field accessory using a 20× objective, Ophir, Darmstadt, Germany). The figure shows the mode profiles of the fundamental modes recorded for each waveguide, indicating that the light remains confined in the waveguide core without leaking into a possible residual layer. Furthermore, for the waveguides with a width equal or below 3 µm, no higher order modes could be excited, indicating the single mode behavior of these waveguides.

### 4.2. Imprinted Bragg Grating Sensors

**Figure 7:**
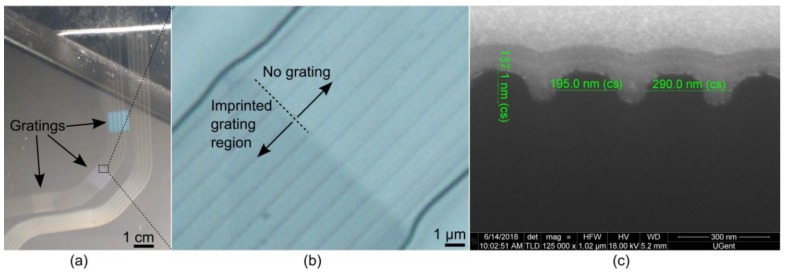
(**a**) Gratings imprinted in the waveguide core layer, visualized before applying the top cladding layer; (**b**) a magnified view on the diagonally oriented grating; (**c**) Imprinted OrmoCore grating cross-section.

The macro photo shown in Figure 7 gives a first impression of imprinted Bragg gratings and their orientation and position along the waveguide length. Note that several arrays of parallel waveguides were fabricated on a single sample for the purpose of process optimization; for the functional sensor tests as discussed in Section 4.2 and Section 4.3, a single waveguide with cross-sectional dimensions 3 × 3 µm^2^ was used. The quality of the Bragg gratings imprinted at the waveguide core-upper cladding interface was further investigated using microscopy and Field Emission Gun Scanning Electron Microscopy (FEG SEM). Figure 7b shows a magnified view of the waveguides, where the imprinted grating section starts. It can be seen that the presence of the grating did not prevent the waveguide from capillary filling during the imprinting process. The waveguides are nicely formed with a smooth transition at the grating region which is important to avoid additional optical loss. Finally, Figure 7c, shows a focused ion beam cross-section of the imprinted Bragg grating, imaged using a FEG SEM. To facilitate this process, the cross-section was made of a grating fabricated on a separate silicon substrate using the same soft stamp and optical polymer (OrmoCore) as used for fabricating the functional sensor foils. The micrograph shows grating features with the expected pitch and thickness (although slightly larger than the targeted 120 nm), but smoother features due to the very challenging e-beam fabrication step for such small feature sizes. This cross-section shows the successful fabrication of the first order Bragg gratings, although we have to mention that defects were visible at some locations when inspecting the grating top surface (see Supplementary material, Figure S1). We suspect that these defects were formed due to non-perfect replication of the PMMA grooves on the master mold into the PFPE material during the soft stamp fabrication. This replication process can be improved by transferring the e-beam written resist features into the silicon substrate of the master mold using reactive ion etching together with the use of an anti-stick layer applied on the silicon nanostructures before applying the PFPE material; a process which is now being developed. 

**Figure 8:**
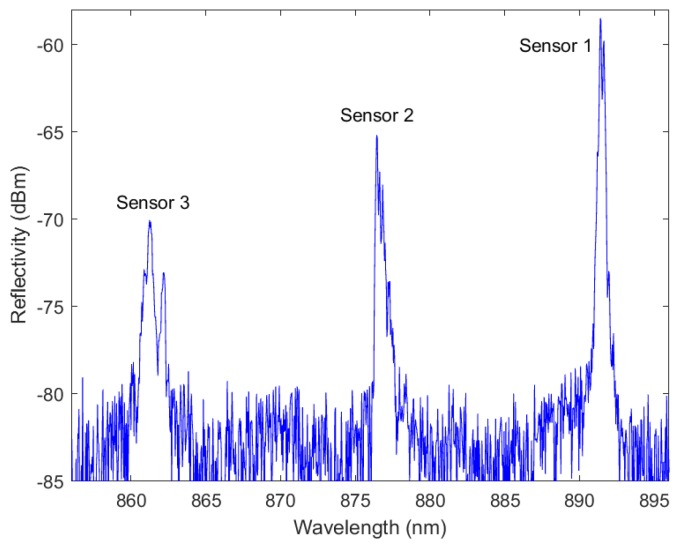
Reflection spectrum of the sensor foil showing the Bragg wavelength of the three sensors multiplexed in the same waveguide.

The reflection spectrum of the sensor was recorded using an OSA, see Figure 8. Three clear peaks can be observed with a spectral separation of about 15 nm as designed, although the spectral shape slightly differs from what is simulated most likely because of the presence of some defects in the imprinted gratings, as discussed above. Although the 3 gratings themselves theoretically have the same reflectivity, the peaks corresponding to the sensors positioned farther away from the connector are weaker due to the increased optical propagation loss in the waveguide. 

### 4.3. Strain Sensitivity

**Figure 9:**
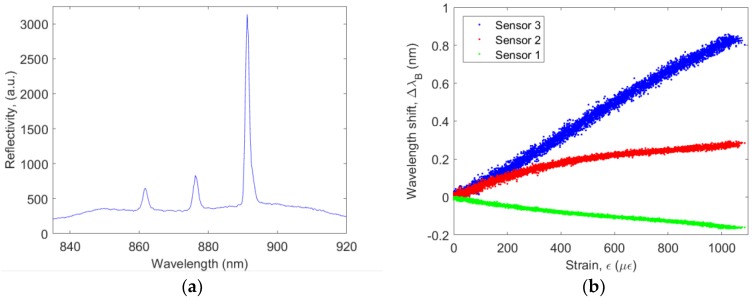
(**a**) Typical reflection spectrum recorded by the readout system. (**b**) Resulting Bragg wavelength as a function of applied strain.

Figure 9 shows a typical sensor reflection spectrum as recorded by the readout system and the resulting wavelength shift recorded for the three sensors as a function of the strain measured by the extensometer. As expected, the sensor which is oriented along the loading direction shows the highest sensitivity, i.e., 0.85 pm/µε. Since the sensitivity of a Bragg grating sensor scales with the operating wavelength, it is convenient to consider the wavelength normalized sensitivity, i.e., (0.85 pm/860 nm)/µε or 0.99 ppm/µε. This result is in line with previous experiments, in which the sensitivity of a similar Bragg grating sensor, but operating at 1540 nm, was determined to be 1.4 pm/µε, or 0.91 ppm/µε [19]. The wavelength normalized strain sensitivity of a typical commercially available silica-based optical fiber Bragg grating sensor is slightly lower, i.e., 0.77 ppm/µε [23].

The sensor oriented perpendicular to the loading direction shows a negative wavelength shift with a slope of −0.14 pm/µε because it is loaded in compression due to the Poisson effect. However, taking into account the Poisson’s ratio of aluminum (0.33), a slope of −0.33 × 0.85 pm/µε = −0.28 pm/µε would be expected.

**Figure 10:**
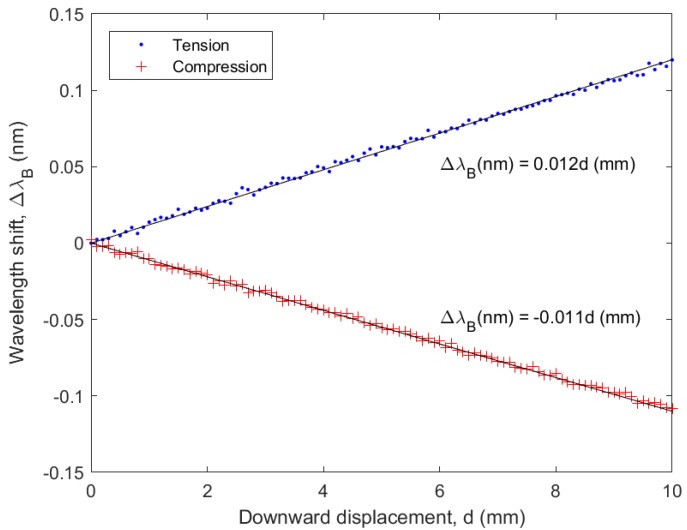
Bragg wavelength shift recorded for sensor 3, in tension and in compression during the cantilever loading experiment.

This discrepancy can be due to either a different sensor sensitivity in compression as compared to tension, or due to non-ideal sensor attachment or bending of the aluminum dogbone test specimen in the direction perpendicular to the loading direction, because it was rather thin. The results of the cantilever test however, as shown in Figure 10, show that the sensors behave very similarly in tension and compression. The unexpected signal of the perpendicularly oriented sensor during the tensile test is therefore likely caused by non-ideal testing conditions, and not due to the sensor operation itself. This is furthermore supported by the nonlinearity in the graph in Figure 9b and will be confirmed in future experiments, e.g., by performing a different type of loading experiment such as a four point bending test. A demonstration of the multi-axial sensing capability of the foils is given in Video S2 in which the wavelength shift experienced by the three Bragg grating sensors is shown in real time when bending the free-standing sensor foil. The sensor oriented along the bending direction shows the largest change in signal, while the sensor oriented under 45° shows roughly half the signal change, and the perpendicularly oriented sensor shows virtually no change. 

A remark needs to be made regarding the resolution of the USB spectrometer used. It can be seen that peaks in the spectrum recorded by the Ocean Optics spectrometer (Figure 9) are smoother and broader than those recorded using the OSA (Figure 8). This is due to the limited spectral resolution of the general purpose spectrometer that was available (resolution = 1.4 nm) and can easily be improved by using a dedicated spectrometer optimized for working in the sensor operation wavelength range. It has to be mentioned however, that the peak detection resolution in our experiment was much better than the spectrometer resolution, since instead of a simple maximum detection, the peaks in the recorded spectra were fitted to Gaussian peaks of which the center of mass was determined. Finally, the sensor 2 and sensor 3 signals (Figure 9b) are noisier as compared to the signal from sensor 1. This is due to the considerably lower signal-to-noise ratio of these sensor signals, which limits the accuracy of the measurement. The reason for this is that these grating sensors are positioned farther away from the connector. The longer distance that the optical signals need to travel, together with the bend sections that need to be bridged, results in more optical losses, leading to reduced signal strength.

### 4.4. Temperature Sensitivity

**Figure 11:**
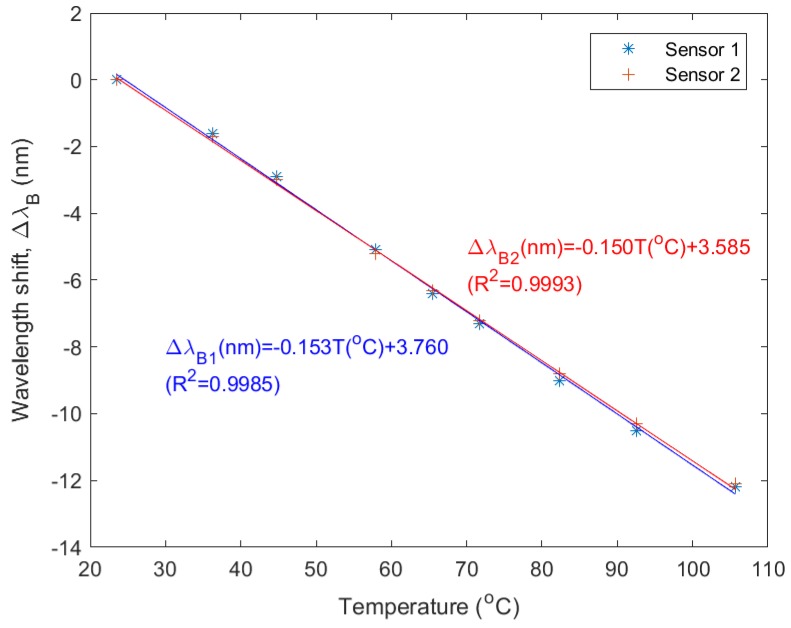
Bragg wavelength shift as a function of temperature for sensor 1 and sensor 2.

Figure 11 shows the Bragg wavelength shift as a function of temperature, for sensor 1 and 2. From the linear fit it can be seen that the sensor sensitivity was found to be −153 pm/°C and −150 pm/°C for sensor 1 and 2 respectively (−172 ppm/°C) in the considered temperature range, which is in line with previous experiments, in which we found a temperature sensitivity of −249 pm/°C for Ormocer^®^-based Bragg grating sensors operating around *λ* = 1540 nm (−162 ppm/°C) [24]. A typical commercially available silica-based optical fiber Bragg grating sensor has a wavelength normalized sensitivity of 6.5 ppm/°C [23], or about 25 times lower and with an opposite sign. This can be explained by the very large and negative thermo-optic coefficient of the used sensor materials. However, it should be mentioned that although the sensitivity of the sensor foils is much higher compared to silica-based sensors, the operating temperature range is much smaller. The Ormocer^®^ sensor materials are in principle stable up to about 200 °C, but the PET foil substrate used will already start softening at lower temperatures.

## 5. Conclusions

A thin polymer sensor foil realized using a stamp-based imprinting technology that can be used as a photonic strain gage rosette or temperature sensor was presented. 

It was shown that the imprinting technology allows definition of very fine functional grating structures (with a pitch around 285 nm) in Ormocer^®^-based hybrid organic-inorganic materials. However, as discussed, the feature definition can be improved by employing a more stable silicon master mold. Furthermore, we have shown that it is possible to realize high-quality imprinted single mode waveguides (cross-sectional dimensions 3 × 3 µm^2^) with a very thin residual layer which is important to limit bend losses or cross-talk with neighboring waveguides.

The strain and temperature sensitivities of the Bragg grating-based sensors, which operate around a wavelength of 850 nm, were determined and compared to the sensitivities of similar Bragg grating sensors operating around 1550 nm. First of all, the obtained 0.85 pm/µε strain and −150 pm/°C temperature sensitivities correspond well with those of previously reported sensors based on the same materials but operating around 1550 nm, taking into account that sensitivity scales with the wavelength. Compared to silica-based fiber sensors, the wavelength normalized strain sensitivity is slightly higher, while the temperature sensitivity is much higher and with opposite sign, due to the very large and negative thermo-optic coefficient of the polymer materials used. Finally, it was shown that multi-axial strain sensing is possible using these sensor foils. This was accomplished by fabricating three closely spaced sensors angularly displaced by 45°, thereby realizing a strain gage rosette. 

## Figures and Tables

**Table 1:** The calculated Bragg wavelength and 3 dB bandwidth for the three gratings with different pitch. The depth, the length and the duty cycle are the same for all gratings.

	Grating Pitch (nm)	Bragg Wavelength (nm)	3dB Bandwidth (nm)
Sensor 3	280	859.6	0.220
Sensor 2	285	874.95	0.231
Sensor 1	290	890.3	0.242

## Supplementary Materials

The following are available online at http://www.mdpi.com/1424-8220/18/8/2717/s1, Figure S1: Top view SEM micrograph of the imprinted grating showing defects, Video S1: Video illustrating the multi-axial sensing behavior of the foil.
