# Supplementary figures and images for: Bragg-Grating-Based Photonic Strain and Temperature Sensor Foils Realized Using Imprinting and Operating at Very Near Infrared Wavelengths
Source: Sensors (Basel). 2018 Aug 18;18(8):2717. doi: 10.3390/s18082717 (PMC6111415; doi:10.3390/s18082717)

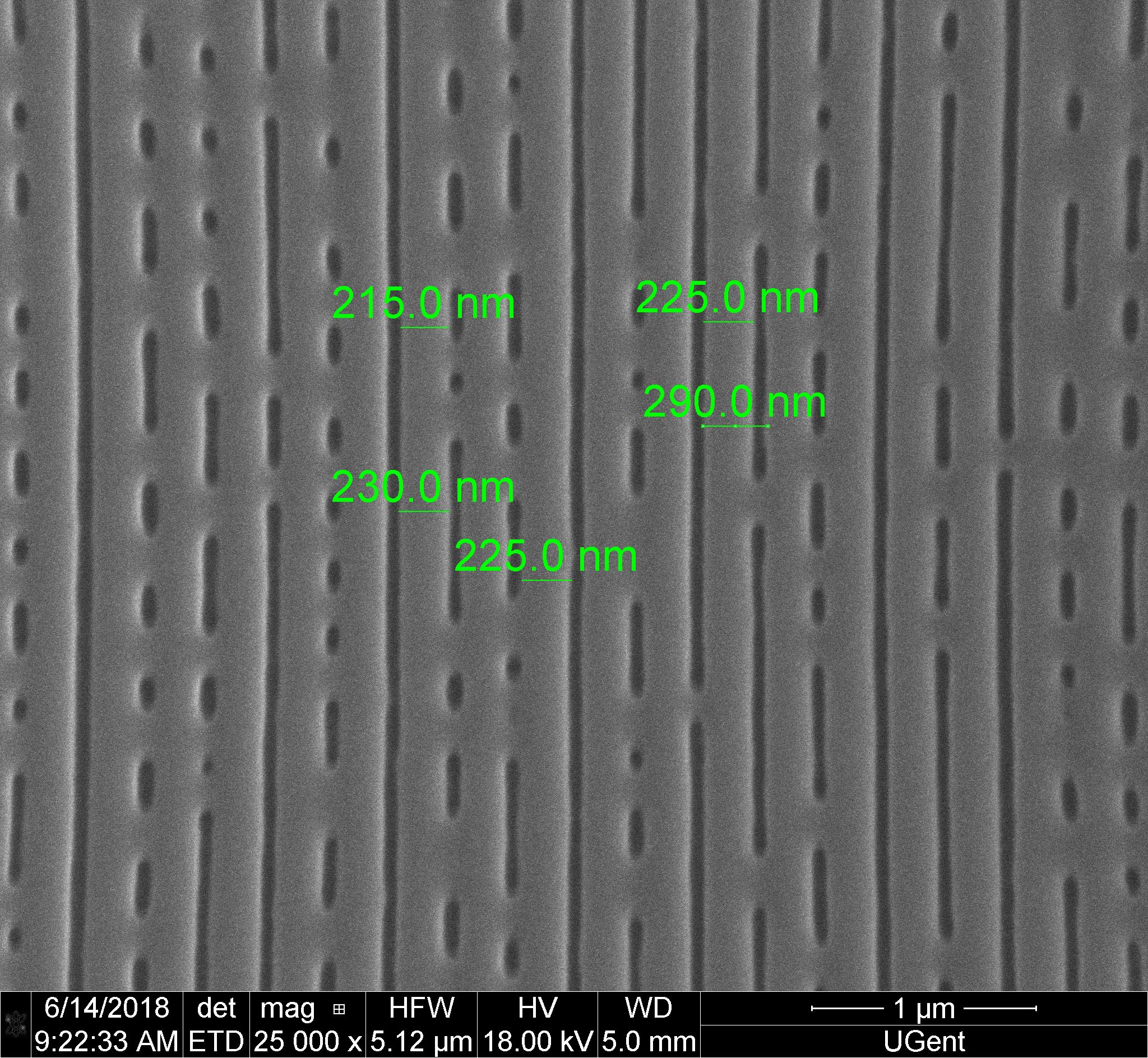

Supplement: Supplementary file 1 [file sensors-18-02717-s001.zip › Figure-S1.jpg]
